# Prediction of pathogenesis-related secreted proteins from *Stemphylium lycopersici*

**DOI:** 10.1186/s12866-018-1329-y

**Published:** 2018-11-20

**Authors:** Rong Zeng, Shigang Gao, Lihui Xu, Xin Liu, Fuming Dai

**Affiliations:** Institute of Eco-Environment and Plant Protection, Shanghai Key Laboratory of Protection Horticultural Technology, Shanghai Academy of Agricultural Sciences, Shanghai, 201403 China

**Keywords:** Tomato leaf gray spot disease, Signal peptide, Effector, Pathogen-host interaction, CAZymes, SCRSPs

## Abstract

**Background:**

Gray leaf spot is a devastating disease caused by *Stemphylium lycopersici* that threatens tomato-growing areas worldwide. Typically, many pathogenesis-related and unrelated secreted proteins can be predicted in genomes using bioinformatics and computer-based prediction algorithms, which help to elucidate the molecular mechanisms of pathogen-plant interactions.

**Results:**

*S. lycopersici*-secreted proteins were predicted from 8997 proteins using a set of internet-based programs, including SignalP v4.1 TMHMM v2.0, big-PI Fungal Predictor, ProtComp V9.0 and TargetP v1.1. Analysis showed that 511 proteins are predicted to be secreted. These proteins vary from 51 to 600 residues in length, with signal peptides ranging from 14 to 30 residues in length. Functional analysis of differentially expressed proteins was performed using Blast2GO. Gene ontology analysis of 305 proteins classified them into 8 groups in biological process (BP), 6 groups in molecular function (MF), and 10 groups in cellular component (CC). Pathogen-host interaction (PHI) partners were predicted by performing BLASTp analysis of the predicted secreted proteins against the PHI database. In total, 159 secreted proteins in *S. lycopersici* might be involved in pathogenicity and virulence pathways. Scanning *S. lycopersici*-secreted proteins for the presence of carbohydrate-active enzyme (CAZyme)-coding gene homologs resulted in the prediction of 259 proteins. In addition, 12 of the 511 proteins predicted to be secreted are small cysteine-rich proteins (SCRPs).

**Conclusions:**

*S*. *lycopersici* secretory proteins have not yet been studied. The study of *S*. *lycopersici* genes predicted to encode secreted proteins is highly significant for research aimed at understanding the hypothesized roles of these proteins in host penetration, tissue necrosis, immune subversion and the identification of new targets for fungicides.

**Electronic supplementary material:**

The online version of this article (10.1186/s12866-018-1329-y) contains supplementary material, which is available to authorized users.

## Background

**Fig. 1:**
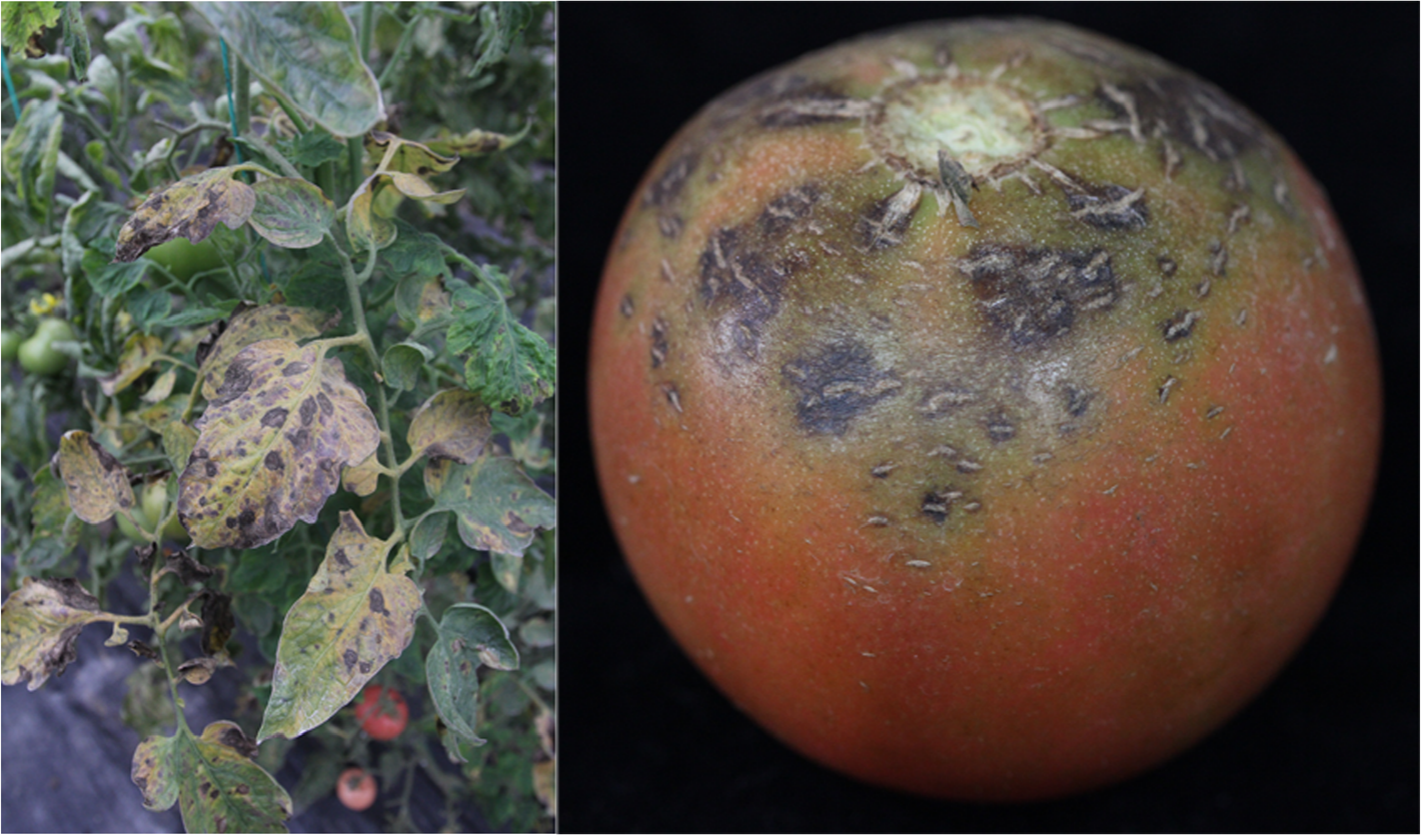
Symptoms of *S. lycopersici* in field on tomato leave and fruit

The fungus *Stemphylium lycopersici* is distributed worldwide and causes gray leaf spots on tomatoes and other crops, resulting in a great decrease in fruit quality and production. On tomato leaves, the disease first appears as circular to elongated dark specks. As the spots enlarge, they become gray or dark brown. Severely infected leaves turn yellow and then die and drop (Fig 1) [1–5].

Various plant pathogens, including fungi, oomycetes, bacteria, and nematodes, contains an ancient and conserved mechanism which secreted proteins and other molecules into cells of the hosts to colonize in plants or against the plant’s immune system [6–9]. Studies of the functions of these secreted proteins and molecules are critical for understanding the mechanisms of potential host colonization and pathogenicity [9]. Interactions between plants and pathogens involve the sensing and secretion of many signal molecules, elicitins and pathogenic factors that interact with plant receptor proteins [6, 7]. A number of avirulence (AVR) proteins, pathogenic factors, and hydrolases are secreted proteins [10–12]. Some elicitins, which function as AVR factors, are a family of structurally related extracellular proteins that induce hypersensitive cell death and other biochemical changes associated with defense responses in plants. For example, *INF1*, *INF2A*, *INF2B*, and *Avr3a* in potato late blight *Phytophthora infestans* [13, 14]; and *Avr-Pita*, *AvrPiz-t* and *PWL2* in rice blast fungus caused by *Magnaporthe oryzae* [8, 15–17] exhibit pathogenic functions during pathogen infection. PsXEG1, a glycoside hydrolase 12 (GH12) from *P. sojae*, is a novel pathogen-associated molecular pattern (PAMP) [18].

The availability of public genomic sequencing data has significantly benefited the research community in the study of fungal genetics, fungal biology, gene function and plant pathology to investigate and control pathogen-host interactions (PHIs). Many scholars have used bioinformatics tools to predict secreted proteins from *Aspergillus nidulans*, *Saccharomyces cerevisiae*, *Agrobacterium tumefaciens*, *Fusarium graminearum*, *M. oryzae*, *Puccinia helianthi*, *P. infestans*, and *Neurospora crassa* [19–23]*.* However, *S. lycopersici*-secreted proteins have not yet been analyzed. In this study, internet-based tools, such as SignalP v4.1, Transmembrane Helices Hidden Markov Model (TMHMM) v2.0, big-PI Fungal Predictor, ProtComp V9.0 and TargetP v1.1, were used to predict typical *S. lycopersici*-secreted proteins, resulting in the identification of 511 proteins predicted to be secreted among 8997 *S. lycopersici* proteins. This research provides important information on the systematic analysis of the *S. lycopersici* elicitins and pathogenic factors to reveal the molecular mechanisms and interactions between *S. lycopersici* and its hosts.

## Methods

### Sequence data and preparation

The *S. lycopersici* proteome was obtained from the National Center for Biotechnology Information (NCBI) database: ftp://ftp.ncbi.nih.gov/genomes/genbank/fungi/Stemphylium_lycopersici/latest_assembly_versions/GCA_001191545.1_ASM119154v1/GCA_001191545.1_ASM119154v1_protein.faa.gz [24]. Secreted proteins were predicted from the N-terminal amino acid (aa) sequences of 8997 proteins.

### Prediction of secreted proteins

The computational secretome should have the following characteristics: (a) an N-terminal signal peptide; (b) no transmembrane domains; (c) no glycosyl phosphatidyl inositol (GPI)-anchor site; and (d) no localization signal predicted to target the protein to the mitochondria or other intracellular organelles. Open reading frames (ORFs) fulfilling these four criteria were included in the set of secreted proteins that we defined as the computational secretome.

In this study, we used five internet-based programs that were selected for their applicability to high throughput analysis and their ability to predict secreted proteins in *S. lycopersici* (Table 1)*.* We queried the *S. lycopersici* ORF set with SignalP v4.1 to identify N-terminal signal peptides, setting the default D-cutoff for SignalP-noTM networks at 0.45 and the D-cutoff for SignalP-TM networks at 0.5 [25]. Next, TMHMM v2.0 was used to predict transmembrane domains with the default parameters [26], and big-PI Fungal Predictor was used to identify potential GPI-anchor sites [20, 27, 28]. ProtComp V9.0 was used to predict the subcellular localization for fungal proteins [29], and TargetP v1.1 was used to identify secretory pathway signal peptide sequences. In this study, custom Python scripts were used to preprocess text, extract sequences from the *S. lycopersici* proteome for each step of prediction and compare sequences with the PHI database.

**Table 1:** The bioinformatics tools adopted for the prediction of secreted proteins from *S. lycopersici*

Prediction algorithms	Objects predicted	References
SignalP v4.1	Signal peptides	http://www.cbs.dtu.dk/services/SignalP/
TMHMM v2.0	Transmembrane domains	http://www.cbs.dtu.dk/services/TMHMM/
big-PI Fungal Predictor	GPI-anchor site	http://mendel.imp.ac.at/gpi/fungi_server.html
ProtComp v9.0	sub-cellular localization	http://www.softberry.com/
TargetP v1.1	localization	http://www.cbs.dtu.dk/services/TargetP/

### Functional annotation of secreted proteins

Gene ontology (GO) classification of the identified proteins was performed using the web-accessible Blast2GO v4.1 annotation system (https://www.blast2go.com/) [30]. The first step in Blast2Go is to search siminar sequences against the NCBI non-redundant (nr) database by Basic Local Alignment Search Tool protein/nucleotide (BLASTp/BLASTn) with an expectation value of 10^− 3^. Next, mapping and annotation were performed on Blast2GO using default parameters. By applying this methodology, the identified proteins will be divided into three main categories which are biological processes (BP), molecular functions (MF) and cellular components (CC) [30]. Briefly, the FASTA sequences of the secreted proteins were uploaded to Blast2GO, and analysis was performed in three steps as follows: 1) BLAST analysis to identify homologous sequences using a BLAST e-value cutoff of 10^− 3^; 2) mapping to retrieve GO terms; and 3) annotation of the sequence to select reliable functions using a GO weight of 50.

### Prediction of pathogenicity-associated secreted proteins

PHI partners were identified by subjecting predicted secreted proteins to BLASTp against the PHI database (E-value: 10^− 10^) [31].

Fungal extracellular carbohydrate-active enzymes (CAZymes) help break down the components of the plant cell wall, such as complex carbohydrates, allowing fungi to access the host and facilitate infection. To identify *S. lycopersici* CAZymes involved in these processes, we applied the dbCAN2 (http://cys.bios.niu.edu/dbCAN2/) with an e-value cutoff of 10^− 10^ for secreted proteins [32].

Small cysteine-rich secreted proteins (SCRSPs) were predicted based on their expected sequence characteristics and typically consist of 20 to 200 aa residues with an N-terminal signal peptide and at least four cysteine residues. Secreted *S. lycopersici* proteins with these characteristics were identified as putative SCRSPs. Searches for conserved domains of SCRSPs were performed using an online tool Conserved Domain Database (CDD; e-value cutoff of 10^− 3^) (https://www.ncbi.nlm.nih.gov/Structure/cdd/wrpsb.cgi) [33].

## Results

### Secreted protein prediction of the *S. lycopersici* proteome

**Fig. 2:**
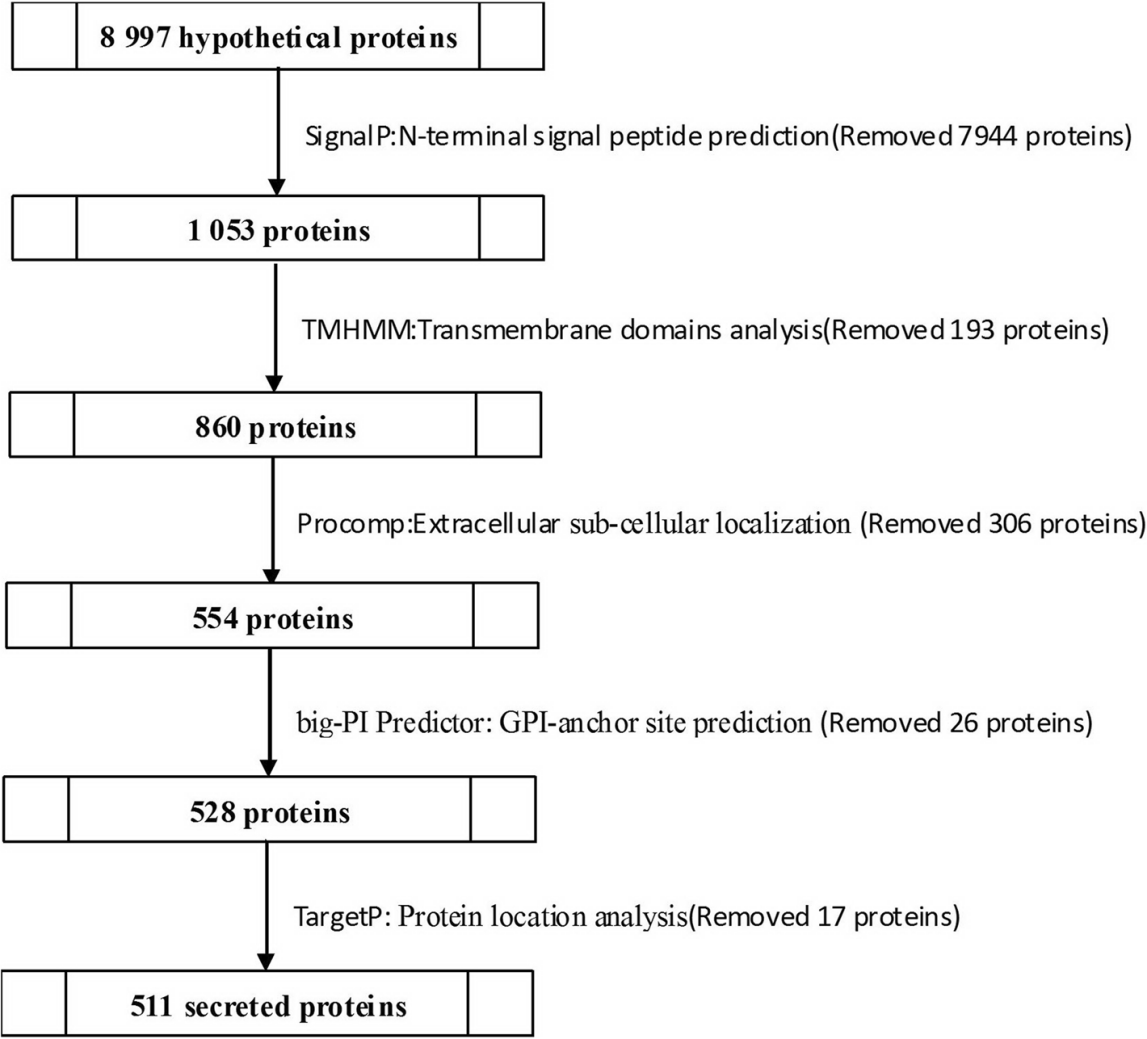
Methods used to predict secreted proteins in *S. lycopersici*

A total of 1053 (11.70%) out of 8997 ORFs were predicted to be classical secreted proteins using SignalP v4.1. The number of transmembrane helices was predicted using TMHMM. Out of the 1053 total predicted secreted proteins, 860 have no predicted transmembrane domain (TMD), and 193 have at least one predicted transmembrane helix. Protcomp v9.0 was used to predict the subcellular localization of the 860 proteins, resulting in 554 extracellular proteins. The big-PI Predictor identified 528 proteins with no GPI modification sites and 26 proteins with one or more GPI-anchored sites. To further confirm that these predicted proteins were secreted from the cell, we performed subcellular localization predictions using TargetP-v1.1. Finally, TargetP v1.1 identified 511 proteins (5.68% of the proteome) that were selected as candidate secreted pathways with signal peptides (Fig 2, Additional file 1).

### Characteristics of secreted proteins and their signal peptides

**Fig. 3:**
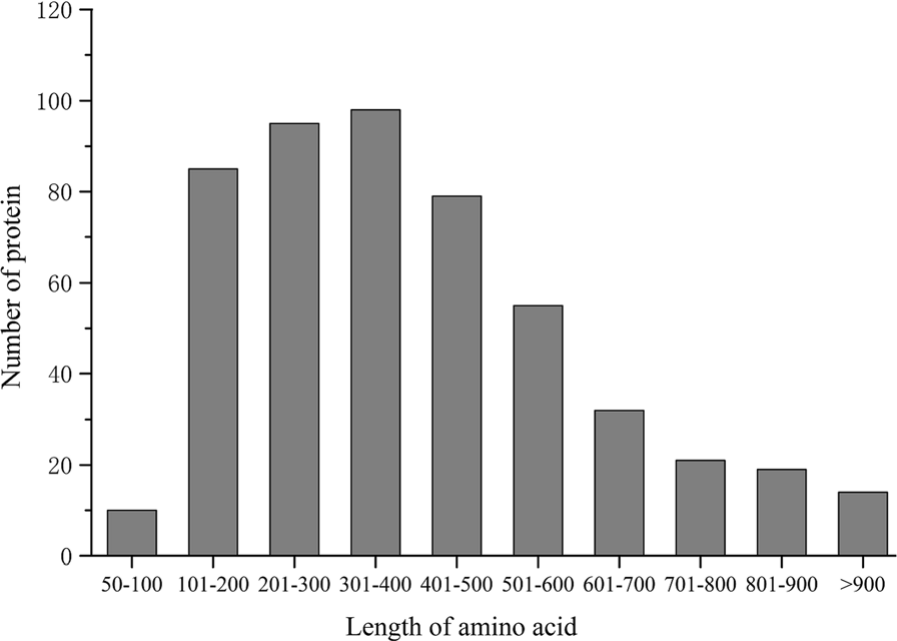
Analysis length of the predicted secreted proteins amino acid residues in *S. lycopersici*

The 511 predicted secreted proteins ranged between 64 and 2260 aa in length, with most (415 proteins) being between 100 and 600 aa in length (81.21% of predicted secreted proteins, mean = 402 aa) (Fig 3).

**Fig. 4:**
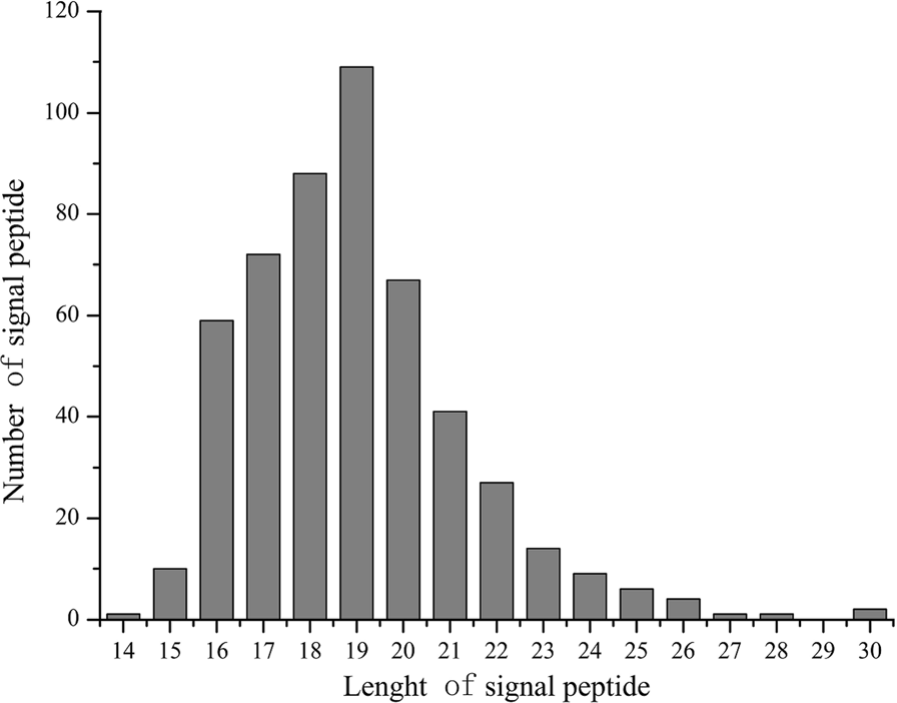
Analysis of the candidate secreted proteins with different length of signal peptide in *S. lycopersici*

The analysis of the signal peptides of the 511 predicted secreted proteins identified signal peptides ranging from 14 to 30 residues in length, with an average of 19 residues. The most common signal peptide length was 19 aa, accounting for 30% of the total (Fig 4).

**Fig. 5:**
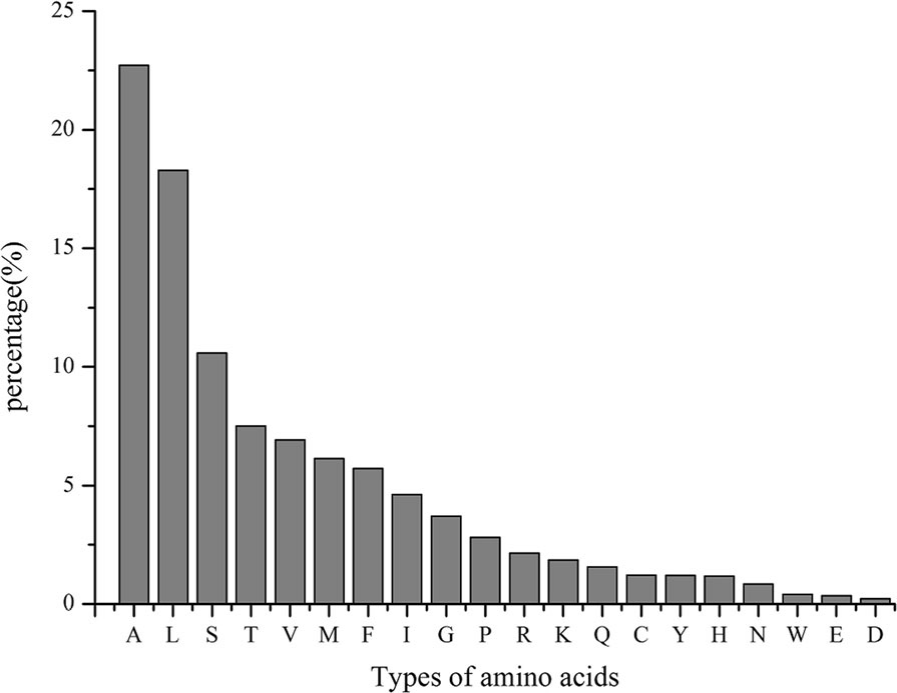
Percentage of 20 amino acid residues in *S. lycopersici* candidate secreted protein signal peptides

The abundances of 20 aa in the signal peptides were analyzed, and the frequencies of these aa (in descending order) were A-L-S-T-V-M-F-I-G-P-R-K-Q-C-Y-H-N-W-E-D. Alanine (A) was the most abundant aa (22.71%) in the assayed signal peptides, followed by leucine (L) (18.29%), while aspartate (D) was the least common (0.22%). Statistical analysis of the aa in the signal peptides showed that nonpolar hydrophobic aa residues (A, L, V, I, G, and P) represented 57.08% of aa, polar noncharged aa residues (S, T, M, Q, C, and N) represented 27.85% of aa, positively charged aa residues (R, K, and H) represented 5.18% of aa, aromatic aa residues (W, F, and Y) represented 7.34% of aa, and negatively charged aa residues (D and E) represented only 0.56% aa (Fig 5).

All aa in the signal peptide cleavage sites were analyzed using a custom Python2.7 script. We defined the three aa N-terminus to the cleavage sites as − 3, − 2, − 1 and the three aa C-terminus to the cleavage sites as + 1, + 2, + 3. The aa with the highest probability of being located at sites between − 3 and 3 were A, S, A, A, P, and T; the frequencies of these aa were 49.32, 20.35, 83.37, 24.07, 35.62, and 12.92%, respectively. An A-S-A motif was most likely to occupy the − 3 to − 1 site and accounted for 51 motifs observed at this site. An A-X-A motif, a typical signal peptidase I (SPase I) cleavage site [34, 35], accounted for 216 motifs (Table 2). SPase I is a serine protease with a catalytic dimer of serine lysine or serine histidine at the active site. The recognition site of SPase I for signal peptide cleavage is determined mainly by an A-X-A motif at the C-terminal of the signal peptide from the cleavage site [35].

**Table 2:** Amino acids frequency and distribution in cleavage site of signal peptide of the predicted secreted proteins in *S. lycopersici*

Kinds of	−3		−2		−1		1		2			3
Amino acid	No.	pct.(%)	No.	pct.(%)	No.	pct.(%)	No.	pct.(%)	No.	pct.(%)	No.	pct.(%)
A	252	49.32%	72	14.09%	426	83.37%	123	24.07%	29	5.68%	44	8.61%
V	133	26.03%	21	4.11%	0	0.00%	20	3.91%	30	5.87%	47	9.20%
T	48	9.39%	35	6.85%	6	1.17%	28	5.48%	42	8.22%	66	12.92%
S	33	6.46%	104	20.35%	28	5.48%	47	9.20%	34	6.65%	49	9.59%
I	20	3.91%	15	2.94%	0	0.00%	18	3.52%	8	1.57%	48	9.39%
G	11	2.15%	6	1.17%	38	7.44%	23	4.50%	21	4.11%	17	3.33%
C	7	1.37%	10	1.96%	5	0.98%	4	0.78%	5	0.98%	11	2.15%
L	5	0.98%	92	18.00%	0	0.00%	36	7.05%	22	4.31%	51	9.98%
H	1	0.20%	16	3.13%	0	0.00%	42	8.22%	7	1.37%	14	2.74%
R	1	0.20%	6	1.17%	0	0.00%	17	3.33%	5	0.98%	13	2.54%
D	0	0.00%	5	0.98%	0	0.00%	7	1.37%	29	5.68%	17	3.33%
E	0	0.00%	15	2.94%	0	0.00%	9	1.76%	13	2.54%	6	1.17%
F	0	0.00%	33	6.46%	0	0.00%	15	2.94%	9	1.76%	25	4.89%
K	0	0.00%	1	0.20%	0	0.00%	10	1.96%	5	0.98%	6	1.17%
M	0	0.00%	12	2.35%	0	0.00%	7	1.37%	8	1.57%	6	1.17%
N	0	0.00%	21	4.11%	0	0.00%	14	2.74%	26	5.09%	18	3.52%
Q	0	0.00%	30	5.87%	3	0.59%	76	14.87%	18	3.52%	20	3.91%
W	0	0.00%	1	0.20%	0	0.00%	7	1.37%	5	0.98%	6	1.17%
Y	0	0.00%	16	3.13%	0	0.00%	8	1.57%	13	2.54%	10	1.96%
P	0	0.00%		0.00%	5	0.98%	0	0.00%	182	35.62%	37	7.24%

*pct* percentage, *A* alanine, *C* cysteine, *D* aspartic acid, *E* glutamic acid, *F* phenylalanine, *G* glycine, *H* histidine, *I* isoleucine, *K* lysine, *L* leucine, *M* methionine, *N* asparagine, *P* proline, *Q* glutamine, *R* arginine, *S* serine, *T* threonine, *V* valine, *W* tryptophan, *Y* tyrosine

The aa residues at the − 3 to + 3 sites were compared between *S. lycopersici* and other plant pathogens. Alanine exhibited the highest frequency of aa residues at the − 3, − 1 and + 1 sites, which are relatively conserved in most species. The *S. lycopersici* aa residues at − 3 to + 3 of the secreted proteins were exactly the same as those in two *Pleosporaceae* pathogens, *Curvularia lunata* and *Cochliobolus heterostrophus* (Table 3) [36]. These conserved residues are important for the recognition and cleavage of the signal peptide.

**Table 3:** The highest frequency of amino acid residue around the cleavage site of signal peptide in *S*. *lycopersici* and other organisms [36]

Organisms	−3	−2	−1	+ 1	+ 2	+ 3
*S*. *lycopersici*	A	S	A	A	P	T
*Curvularia lunata*	A	S	A	A	P	T
*Cochliobolus heterostrophus*	A	S	A	A	P	T
*Agrobacterium tumefaciens*	A	L	A	A	D	L
*Phytophthora infestans*	A	A	A	A	A	A
*Neurospora crassa*	A	L	A	A	P	–
*Verticillium dahliae*	A	S	A	A	P	L
*Ctenopharyngodon idellus*	A	N	A	A	S	S/W
*Caenorthaditis elegans*	V	S	A	Q	P	I
*Puccinia helianthi*	V	S	A	Q	P	L

-: No statistics

### Annotation and classification of *S. lycopersici*-secreted proteins

**Fig. 6:**
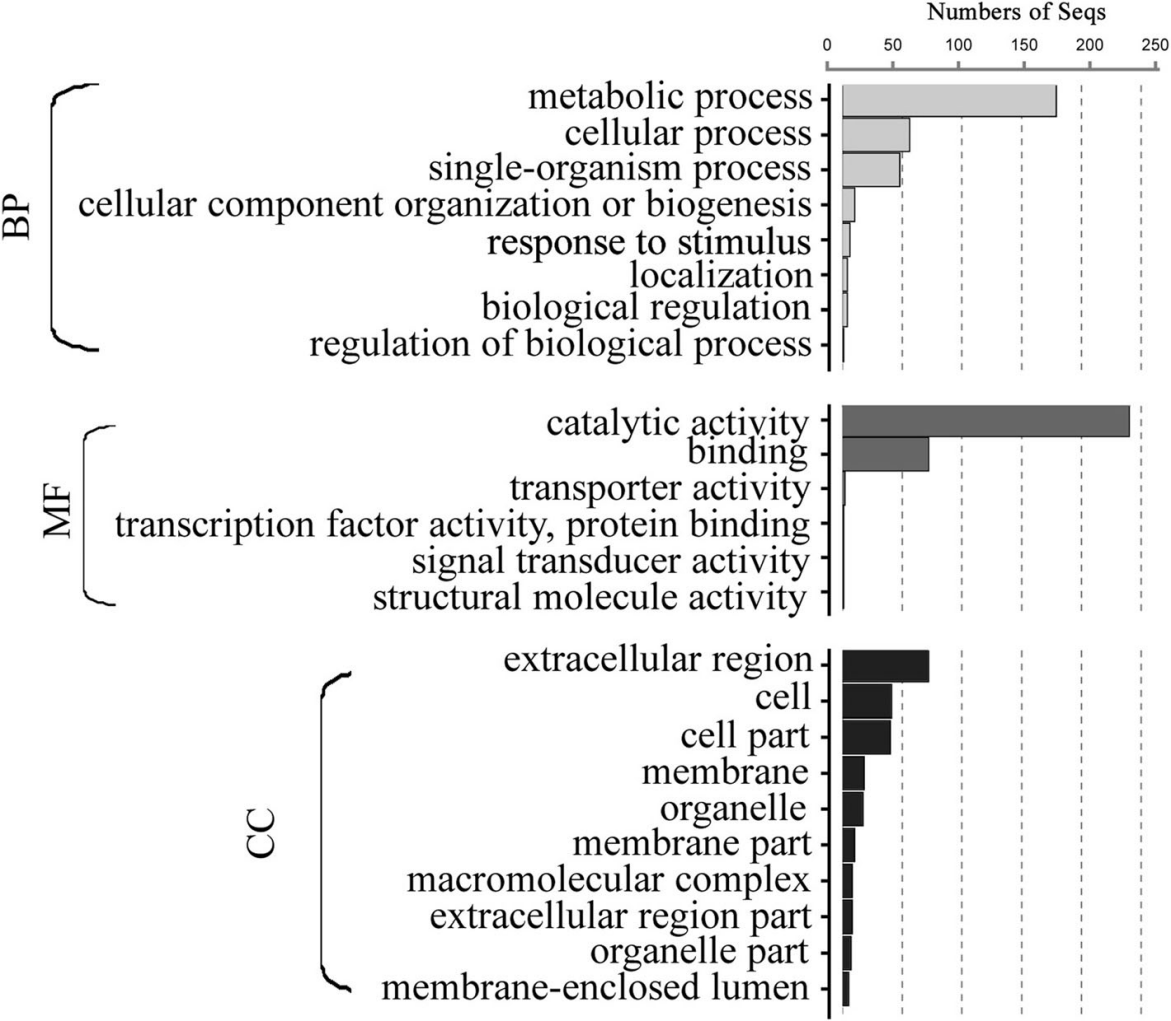
Gene ontology (GO) annotation of the predicted secreted proteins of *S. lycopersici*. The best 305 identified proteins hits were aligned to the GO database and assigned to GO term

Blast2GO is an all-in-one platform for high-quality protein functional prediction and and the genome-wide analysis of annotation data. Using Blast2GO, the 305 identified proteins were potentially classified by their 8 BP groups, 6 MF groups, and 10 CC groups. The functional annotation for the secreted proteins illustrated the following: 1) most representative biological processes—the following categories were highly represented: metabolic processes (GO: 0008152, 179), cellular processes (GO: 0009987, 56) and single-organism processes (GO: 0044699, 48); 2) most representative molecular functions—the following categories dominated: catalytic activity (GO: 0003824, 240) and binding activity (GO: 0005488, 72); and 3) most representative cellular components—the following categories were represented: extracellular region (GO:0005576, 72), cell (GO:0005623, 41) and cell part (GO:00444464, 40) (Fig 6, Additional file 2).

### Pathogenicity-associated secreted proteins

**Fig. 7:**
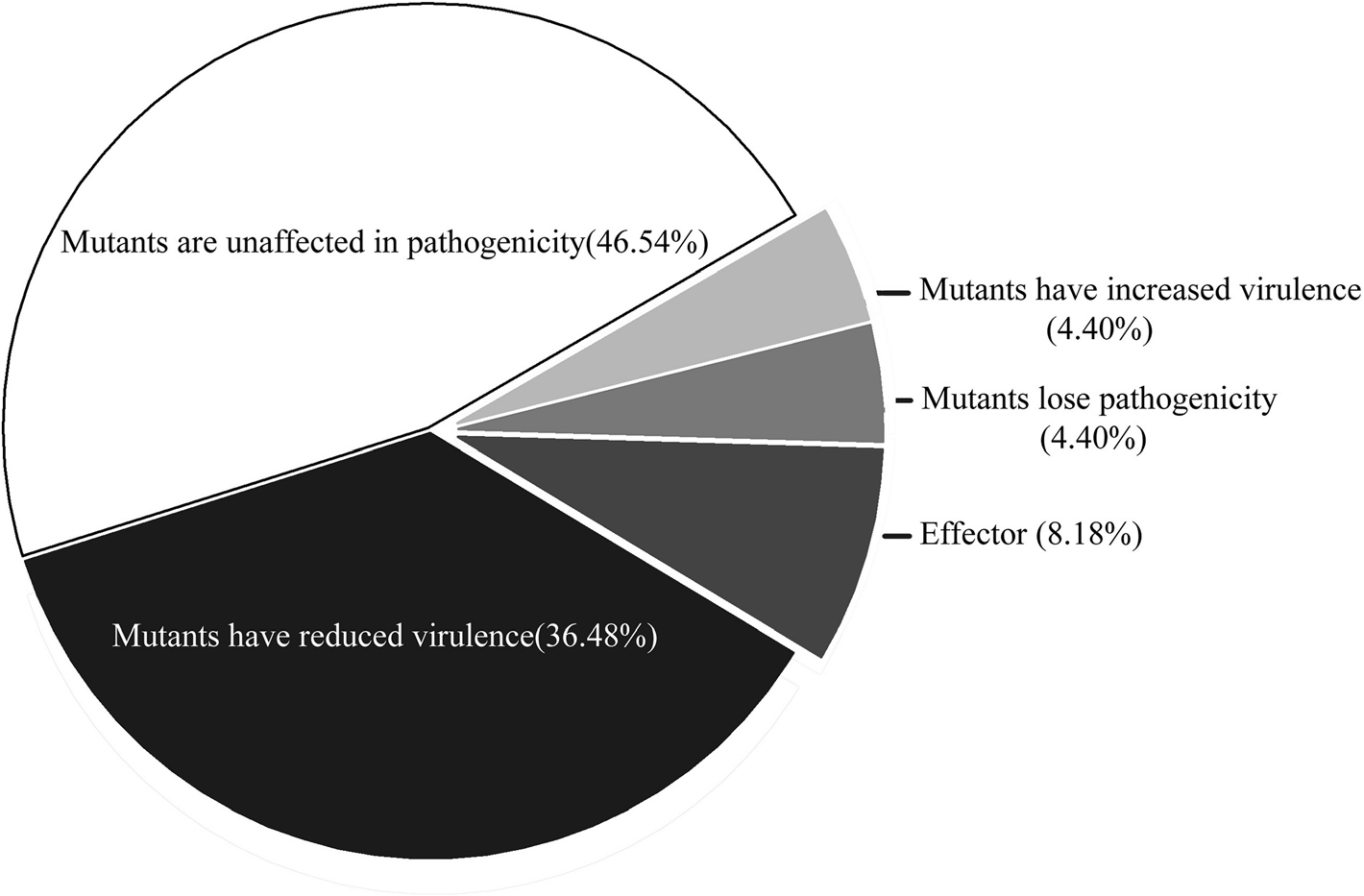
Predicted PHI proteins of the candidate secreted proteins of *S. lycopersici*

The identification of pathogenic-related genes is important to understand the mechanisms of PHIs. According to PHI-base catalogs, 4775 genes and 8610 interactions were predicted to be involved in pathogenicity. In our analysis, a search against PHI-base predicted 159 secreted proteins in *S. lycopersici* that may be involved in pathogenicity and virulence pathways. Of these proteins, 74 secreted proteins could be correlated with “pathogenicity”, 58 secreted proteins were predicted to “reduce virulence”, 13 secreted proteins were predicted to be an “effector”, 7 secreted proteins were predicted to result in “loss of pathogenicity”, and 7 secreted proteins were predicted to result in “increased virulence” (Fig 7, Additional file 3).

Scanning *S. lycopersici*-secreted proteins for the presence of CAZyme-coding gene homologs resulted in the prediction of a set of 259 sequences. The glycoside hydrolase (GH) superfamily was the most highly represented, containing 98 homologs distributed among 38 families. Glycosyl transferases (GT), polysaccharide lyases (PL), carbohydrate esterases (CE), carbohydrate-binding modules (CBM) and auxiliary activities (AA) superfamilies had 1, 22, 40, 42 and 56 homologs each, representing 1, 5, 8, 17 and 6 families, respectively (Table 4, Additional file 4). Comparing our data with those from two other tomato pathogens, *Phytophthora parasitica* and *P. infestans*, demonstrated the variation in CAZyme annotation. In addition to the GT, in terms of the numbers of GH, PL, CE, CBM, and AA CAZyme families, the ratios were quite similar.

**Table 4:** A summary of the carbohydrate-active proteins predicted in *S. lycopesici* and comparison of these data with those from *P. parasitica* and *P. infestans*

	*S. lycopesici*		*P. parasitica*		*P.infestans*	
	CAZyme families	CAZyme modules	CAZyme families	CAZyme modules	CAZyme families	CAZyme modules
GH	38	98	37	293	36	275
GT	1	1	29	169	30	158
PL	5	22	3	47	5	67
CE	8	40	12	113	12	90
CBM	17	42	14	85	16	74
AA	7	56	4	43	8	42

Twelve potential SCRSPs were predicted among the secreted proteins, ranging from 90 to 170 aa residues in size (Table 5). Of these SCRSPs, 8 were annotated in GenBank and had important functions in S. lycopersici. KNG49607, KNG48427 and KNG47745 were annotated as “hypothetical proteins”, and we observed that some of these SCRSPs had common in fungal extracellular membrane proteins (CFEM) domains or lysine motif (LysM) domains [33].

**Table 5:** conserved domains search of the predicted small cysteine-rich secreted proteins (SCRSPs) against CDD database

Gene code	Hit type	Length	Math site	E-Value	Accession	Description of SCRSP
KNG51615.1	specific	156	28–151	8.60E-49	cd12798	alt_a1 major allergen
KNG51272.1	superfamily	138	38–137	2.27E-54	cl03937	polysaccharide lyase family 3 protein
KNG49607.1	specific	90	21–79	0.000154	pfam05730	hypothetical protein TW65_03780
KNG48427.1	specific	153	46–116	2.38E-05	pfam05730	hypothetical protein TW65_04690
KNG46663.1	specific	143	42–139	4.84E-43	cd00606	guanyl-specific ribonuclease f1
KNG46057.1	superfamily	170	73–155	4.97E-07	cl09109	snoal-like polyketide cyclase family protein
KNG45713.1	specific	147	6–128	5.00E-53	COG1956	gaf domain nucleotide-binding protein
KNG50949.1	specific	154	44–147	1.45E-36	cd00448	translation initiation inhibitor
KNG48201.1	superfamily	141	22–140	5.36E-65	cl06331	eliciting plant response protein
KNG47345.1	superfamily	160	24–153	5.19E-31	cl17157	major allergen alt
KNG47745.1	specific	36	51–86	1.75E-07	cd00118	hypothetical protein TW65_05484
KNG46314.1	specific	101	29–89	1.04E-20	pfam06766	hypothetical protein TW65_86951

## Discussion

In nature, plant pathogens have evolved quite distinct and specialised strategies for attacking plants. Many pathogens secrete a number of proteins to facilitate infection by interfering with host cellular functions and by inducing host responses [37–40]. It is of great importance to study the quantity, type and characteristics of secreted proteins in pathogens. Advances in genomic information have provided great opportunities to identify putative secreted proteins in different fungal species.

Based on the 8997 ORFs in the *S. lycopersici* protein database, 511 (5.68%) proteins were predicted to be secreted using a set of bioinformatics tools. These putative secretory proteins were small proteins, and most were proteins of 100 to 600 aa with signal peptides of 16 to 21 aa. The highly conserved signal peptide length distribution suggested that their function is mediated by small differences in the type and sequences of the aa residues.

The abundances of 20 aa in *S. lycopersici* signal peptides were highly similar to those reported in several other pathogenic fungi, including *C. lunata*, *Verticillium dahliae*, *Saccharomyces cerevisiae*, *P. infestans*, and *A. tumefaciens* [19, 21, 36, 41]. Three aa, A, L, and S, were highly represented in the signal peptides. Numerous hydrophobic aa were present in the signal peptides of the putative secreted proteins. This kind of motif may be related to the characteristics of secreted proteins that facilitate signal peptide transport across the membrane [35]. Four major classes of amino-terminal signal peptides can be distinguished on the basis of the SPase recognition sequence. This sequence can help transport proteins to different parts of the cell. Thus, the aa sequence of the cleavage site is essential for SPase recognition. In this study, these sequences included 216 proteins that have a potential signal peptide with a SPase I cleavage site with an A-X-A motif. SPase I, also known as the leader peptidase (Lep), is essential for cell viability, and SPase deficiency results in the accumulation of precursors of secreted proteins [42, 43]. Although the cleavage sites were conserved, the signal peptides were highly evolved. The analysis showed that all 511 signal peptides were not identical in the aa sequence (data not shown), suggesting that each signal peptide may have specific functions.

BLAST2GO is a bioinformatics platform for high-quality functional annotation and analysis of genomic datasets. This program allows for analysis and visualization of newly sequenced genomes by combining state-of-the-art methodologies, standard resources and algorithms [30]. The large number of observed “metabolic process” proteins indicated that these secreted proteins might participate in metabolic processes that include both biosynthetic and catabolic processes. “Catalytic activity” and “binding activity” were the most represented stress-responsive categories, thus indicating that metabolic adjustments may be involved in the PHI process.

PHI-base catalogs experimentally verified pathogenic, virulence and effector genes into a web-accessible database [7, 8, 31]. This database can be used to find novel pathogenic genes in important pathogens, which may be potential targets for fungicides [31]. We predicted 159 PHI-related proteins using BLASTp. Thirteen genes were annotated as an “effector” using BLASTp with PHI-base, and the “effector” was reportedly required for direct or indirect recognition of a pathogen only in the resistant host genotype, which possesses the corresponding disease resistance gene [44]. Some fungal effectors were identified that directly and specifically contributed to eliciting immune responses, perturbing host cellular processes and causing programmed cell death [13, 37, 39, 45].

Plant pathogens may initially use cell wall-degrading enzymes to digest the surface layers of cell walls to facilitate penetration [46, 47]. CAZymes, which are grouped into six functional classes (GH, GT, PL, CE, CBM and AA), are involved in the biosynthesis and degradation of glycoconjugates and oligo- and polysaccharides. In addition, CAZymes play a central role in the synthesis and breakdown of the plant cell wall [46, 48]. The results of the analysis of *S. lycopersici* classical secretory proteins showed that 259 secretory proteins are predicted as CAZymes, accounting for 50.68% of the total secreted proteins. The GH, PL, and CE superfamilies, which accounted for 31.31% of the total secreted proteins, are also known as cell-wall-degrading enzymes (CWDE) due to their role in the disintegration of the plant cell wall by bacterial and fungal pathogens. Given the complexity of carbohydrate biochemistry and the broad range of hydrolytic activities involved in this process, it is unsurprising that the examined genome exhibits a considerable number of GHs, which have extremely detailed enzyme entries in the database [48, 49]. *S. lycopersici*-secreted proteins were especially rich in family GH5 protein models (33 homologs), which act on β-linked oligo- and polysaccharides and glycoconjugates [32, 49]. Most of these CAZymes were unequivocally involved in the biochemical pathways aimed at maintaining fungal metabolism.

Fungal effector proteins are typically small in size. Hydrophobins, small and cysteine-rich hydrophobic proteins, assemble on the surface of hyphae and are required as effectors by pathogens that attach to hydrophobic surfaces [50]. SCRSPs are secreted directly into host plant cells and perform multiple biological functions, such as host recognition or colonization, hypersensitive response (HR) induction and pathogenicity. In this study, we predicted 12 SCRSPs from the *S. lycopersici* secretome. These SCRSPs contain CFEM domains, which typically contain eight cysteine residues, and are fungal-specific extracellular membrane proteins, such as Pth11p of *M. grisea*. Pth11p plays important roles in appressorium formation and fungal pathogenesis [24]. Therefore, hydrophobins or SCRSPs predicted in *S. lycopersici* also likely have key functions in pathogenesis and serve as important candidate proteins for the study of PHI mechanisms.

## Conclusion

In conclusion, bioinformatics tools have been widely applied in molecular biology experiments, promoting the investigation and selection of genes or proteins of interest. Many bioinformatics tools are very efficient at predicting the secretion of proteins in fungi. With the development of next-generation sequencing technology, substantial amounts of plant pathogenic fungal, bacterial and other genomic data have been released. However, *S. lycopersici* secretory proteins have not yet been studied. The release of the *S. lycopersici* whole genome sequence provided some important data for studying the pathogenic factors of *S. lycopersici*. The study of *S. lycopersici* genes predicted to encode secreted proteins is highly significant for research aimed at understanding the potential roles of these proteins in host penetration, tissue necrosis, immune subversion and the identification of new targets for fungicides.

## Additional files

Additional file 1: Sequences of 511 predicted secreted proteins. (FASTA 249 kb)

Additional file 2: Blast2go details. (XLSX 14 kb)

Additional file 3: PHI details. (XLSX 14 kb)

Additional file 4: CAZyme details. (XLSX 74 kb)

## Abbreviations

AVR: Avirulence
BP: Biological Process
CAZymes: Carbohydrate-active Enzymes
CC: Cellular Component
CE: Carbohydrate Esterases
CFEM: Common in Fungal Extracellular Membrane Proteins
GH: Glycoside Hydrolase
GO: Gene Ontology
GPI: Glycosyl Phosphatidyl Inositol
GT: Glycosyltransferases
LysM: Lysine Motif
MF: Molecular Function
ORF: Open Reading Frame
PAMPs: Pathogen-associated Molecular Patterns
PHI: Pathogen-host Interaction
PL: Polysaccharide Lyases
SCRPs: Secreted Small Cysteine-rich Proteins
SPase: Signal Peptidase
TMD: Transmembrane Domain
