# Correction to: Prediction of pathogenesis-related secreted proteins from Stemphylium lycopersici

**DOI:** 10.1186/s12866-018-1385-3

**Published:** 2019-01-21

**Authors:** Rong Zeng, Shigang Gao, Lihui Xu, Xin Liu, Fuming Dai

**Affiliations:** Institute of Eco-Environment and Plant Protection, Shanghai Key Laboratory of Protection Horticultural Technology, Shanghai Academy of Agricultural Sciences, Shanghai, 201,403 China

**Correction to: BMC Microbiology (2018) 18:191.**

**https://doi.org/10.1186/s12866-018-1329-y**

Following publication of the original article [1], we have been notified that the study on molecular mechanisms of pathogen-plant interactions about Stemphylium lycopersici of Dr. Franco was not correctly cited, but solely mentioned in the acknowledgements section. The authors would like to apologize for this omission and to express their appreciation for Dr. Franco’s and co-authors’ excellent work and selfless contribution.

The correct citation for the data presented in section “Materials and Methods - Sequence data and preparation” (ftp://ftp.ncbi.nih.gov/genomes/genbank/fungi/Stemphylium_lycopersici/latest_assembly_versions/GCA_001191545.1_ASM119154v1/GCA_001191545.1_ASM119154v1_protein.faa.gz) is:

[25] Franco ME, Lopez S, Medina R, Saparrat MC, Balatti P: Draft Genome Sequence and Gene Annotation of Stemphylium lycopersici Strain CIDEFI-216. Genome Announc 2015, 3(5).
